# Deep Learning-Based Dental Caries Diagnosis: A Modality-Stratified Systematic Review and Meta-Analysis of Faster R-CNN and Mask R-CNN

**DOI:** 10.3390/diagnostics16050731

**Published:** 2026-03-01

**Authors:** Quang Tuan Lam, Minh Huu Nhat Le, Fang-Yu Fan, Nguyen Quoc Khanh Le, I-Ta Lee

**Affiliations:** 1 School of Dentistry, College of Oral Medicine, Taipei Medical University, Taipei 11031, Taiwan; d204111005@tmu.edu.tw; 2 AIBioMed Research Group, Taipei Medical University, Taipei 11031, Taiwan; d142111009@tmu.edu.tw; 3 Faculty of Odonto-Stomatology, Da Nang University of Medical Technology and Pharmacy, Da Nang 550000, Vietnam; 4 International Ph.D. Program in Medicine, College of Medicine, Taipei Medical University, Taipei 11031, Taiwan; 5 School of Dental Technology, College of Oral Medicine, Taipei Medical University, Taipei 11031, Taiwan; fish884027@tmu.edu.tw; 6 Artificial Intelligence in Medicine, College of Medicine, Taipei Medical University, Taipei 11031, Taiwan; 7 Translational Imaging Research Center, Taipei Medical University Hospital, Taipei 11031, Taiwan

**Keywords:** dental caries, deep learning, diagnostic accuracy, faster R-CNN, mask R-CNN

## Abstract

**Background:** Deep convolutional neural networks (DCNNs) are increasingly used in computer-aided dental diagnostics. However, the relative diagnostic performance of commonly applied architectures, particularly Faster R-CNN and Mask R-CNN, has not been systematically synthesized across imaging modalities. This systematic review and meta-analysis compared the diagnostic accuracy of Faster R-CNN and Mask R-CNN for dental caries detection using radiographic and photographic images. **Methods:** PubMed (MEDLINE), EMBASE, Web of Science, and Scopus were systematically searched for studies published up to 15 June 2025. Studies applying Faster R-CNN and/or Mask R-CNN to dental caries detection were included. Binary diagnostic data were extracted, and pooled sensitivity, specificity, and area under the receiver operating characteristic curve (AUC) were estimated using a bivariate random-effects model. Study quality was assessed with QUADAS-AI, and radiomics-based radiographic studies were additionally evaluated using the Radiomics Quality Score (RQS). The protocol was registered in PROSPERO (CRD420251074443). **Results:** Seventeen studies met the inclusion criteria. Across all imaging modalities, Mask R-CNN showed significantly higher pooled sensitivity (85.6% vs. 71.7%, *p* = 0.0244), specificity (94.2% vs. 81.4%, *p* = 0.00089), and AUC (0.95 vs. 0.84, *p* = 0.0053) than Faster R-CNN. In radiographic images, Mask R-CNN consistently outperformed Faster R-CNN in sensitivity (86.3% vs. 67.2%, *p* = 0.0497), specificity (96.5% vs. 85.0%, *p* = 0.00105), and AUC (0.97 vs. 0.86, *p* = 0.0067). In photographic images, Mask R-CNN achieved a higher AUC (0.91 vs. 0.83, *p* = 0.048), whereas differences in pooled sensitivity (83.5% vs. 77.3%, *p* = 0.435) and specificity (86.0% vs. 75.1%, *p* = 0.156) were not statistically significant. **Conclusions:** Faster R-CNN and Mask R-CNN both show potential for dental caries detection, but current evidence is limited by substantial heterogeneity, predominantly retrospective designs, and variability in imaging and labeling. Across the included studies, Mask R-CNN showed higher pooled performance estimates than Faster R-CNN, with the clearest differences in radiographic applications; however, this comparison is indirect and should be considered suggestive rather than definitive given study-level heterogeneity and uncertainty in the reference standard in a sizable proportion of studies. Prospective, multi-center studies with standardized imaging protocols, rigorous annotation, and independent external validation are required to support reliable clinical implementation.

## 1. Introduction

Growing evidence indicates that oral health is closely linked not only to oral diseases but also to overall health. More specifically, untreated oral diseases have been consistently associated with a range of systemic disorders [1]. According to the Global Burden of Disease Study (GBD), only limited population-level improvement has been observed in oral health outcomes over the past three decades, with relatively small changes in oral condition estimates between 1990 and 2021 [2]. Consequently, oral diseases and the delivery of dental care are increasingly recognized as important public health priorities.

Dental caries is a biofilm-mediated, sugar-driven, multifactorial, dynamic disease resulting in the phasic demineralization and remineralization of dental hard tissues [3]. A 2025 GBD-based analysis estimated that, dental caries affected 2.24 billion individuals with permanent tooth caries and 520 million children with primary tooth caries globally [4]. Early and accurate detection of carious lesions is essential for informing clinical decision-making and optimizing treatment outcomes [5]. Dental caries is currently diagnosed through visual–tactile examination combined with radiographic assessment—a workflow that demands substantial expertise and is time-consuming. Consequently, diagnostic variability and human error can lead to missed or misclassified lesions, underscoring the need for emerging technologies to augment conventional workflows and improve early detection sensitivity [6].

Artificial intelligence (AI) is a domain of applied computer science that employs computational systems to emulate human behaviors such as intelligent reasoning, critical thinking, and decision-making [7]. In recent years, AI has attracted considerable scientific interest for its capacity to transform fields that traditionally depend on manual labor. Medicine is a prominent example, with accumulating evidence supporting AI’s utility as a diagnostic aid [8]. Innovative, technology-enabled diagnostic and therapeutic methods can shorten clinical workflows and anticipate potential adverse events [9]. Deep learning (DL)—a subfield of AI characterized by multilayer neural networks and automatic feature learning—has largely superseded earlier AI techniques and has become the dominant paradigm in healthcare. DL-based developments can function as clinical decision support and provide second-opinion capabilities in routine practice [8]. Convolutional neural networks (CNNs), a class of DL models, have achieved state-of-the-art performance [10] in dental radiologic tasks, including assessment of periodontal bone loss [11], detection of carious lesions [12], segmentation of apical pathology [13], and identification of dental plaque [14].

**Figure 1:**
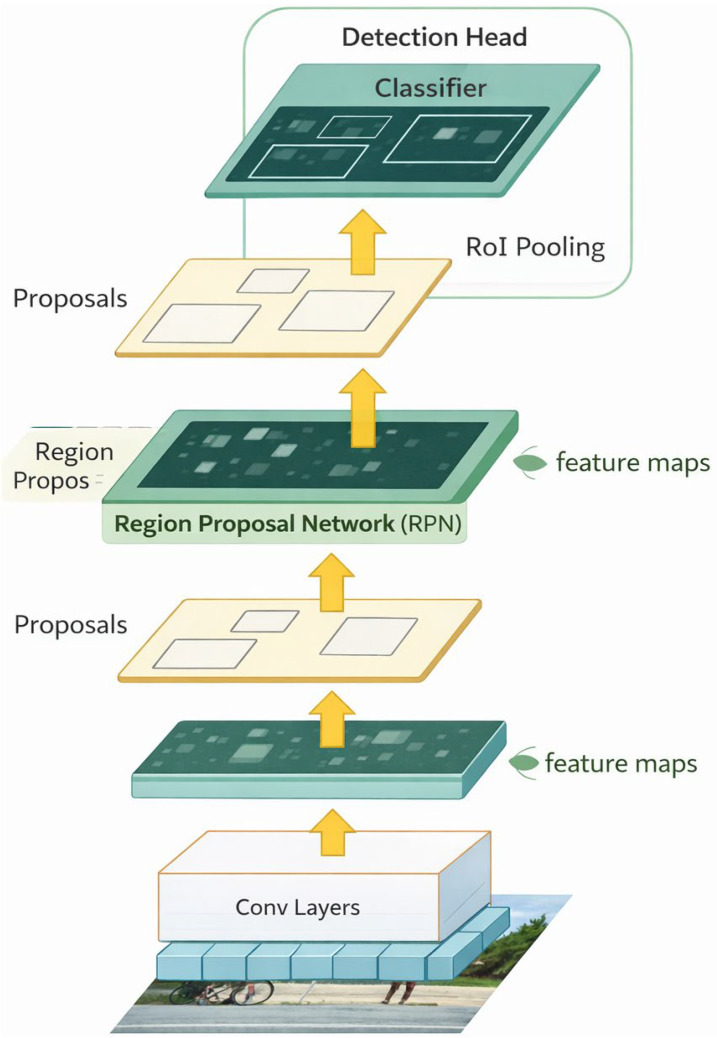
Faster R-CNN architecture diagram.

Faster R-CNN (Figure 1), introduced by Ren et al. at Microsoft Research, is a two-stage object detection framework that unifies region proposal generation and object classification within a single convolutional architecture. A Region Proposal Network (RPN) efficiently generates candidate regions while sharing convolutional features with the detection head, thereby reducing computational cost [15]. By eliminating external proposal methods such as selective search, this design substantially improves speed, particularly on GPUs. Faster R-CNN attains high accuracy and performs well on small objects due to its high-resolution, region-focused processing [16]. It has become a widely adopted baseline in object detection benchmarks such as PASCAL VOC and MS COCO, and its flexibility facilitates adaptation to diverse backbones and application domains, including medical imaging and remote sensing [17]. In dental applications using bitewing radiographs and intraoral photographs, Faster R-CNN is commonly initialized with ResNet-50 or ResNet-101 backbones pretrained on ImageNet and subsequently fine-tuned on domain-specific datasets [18]. The RPN is frequently customized by reducing anchor sizes to 8–32 pixels and adopting aspect ratios of 1:1, 1:2, and 2:1 to better reflect the small-scale morphology of interproximal caries, which differs from natural-image corpora such as MS COCO; high-IoU anchors are then passed to a two-stage classifier and regressor to produce Regions of Interest (ROIs) [19]. Faster R-CNN has seen growing use in dental imaging for tooth detection, numbering, and lesion identification. For example, Mima et al. used zone-specific detectors on panoramic radiographs to detect and classify all 32 permanent teeth, achieving 98.9% sensitivity and 91.7% accuracy while reducing false positives [20]. Similarly, Sari et al. identified dens invaginatus—a rare dental anomaly—on panoramic images with 0.91 precision and 0.90 sensitivity, outperforming YOLOv8 in diagnostic accuracy [21]. These findings underscore Faster R-CNN’s suitability for detecting small, irregular dental structures, a key advantage for clinical diagnostics, and support its integration into computer-aided diagnosis to enhance charting consistency, early pathology detection, and treatment planning.

**Figure 2:**
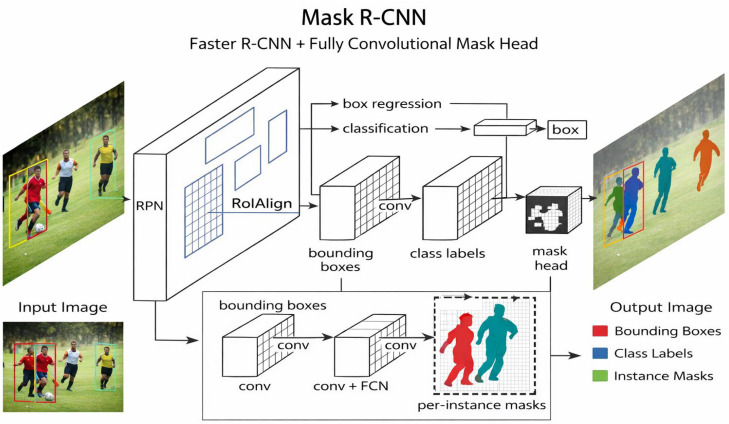
The Mask R-CNN framework for instance segmentation.

Mask R-CNN (Figure 2) extends Faster R-CNN by adding a third, fully convolutional mask head that predicts a binary segmentation map for each Region of Interest (ROI), while RoIAlign preserves sub-pixel alignment; the network therefore outputs bounding boxes, class labels, and per-instance masks in a single pass. Conceptually, it integrates Faster R-CNN’s two-stage detector (RPN plus classifier/regressor) with the encoder–decoder principles of U-Net-style fully convolutional networks, enabling first localization and then high-resolution decoding—an approach well suited to the small, irregular structures common in dentistry [22]. Fatima et al. reduced RPN anchor scales to 8 pixels with aspect ratio multipliers of 0.5:1:2, allowing Mask R-CNN to capture tiny cavitated regions in periapical radiographs; their implementation, based on a MobileNet-v2 + FPN (or ResNet-50) encoder fine-tuned end-to-end for 50 epochs, demonstrated that lightweight backbones can deliver speed without sacrificing accuracy [23]. On CBCT volumes, Ma et al. transferred COCO-pretrained ResNet-50 weights into Mask R-CNN and, after 200–300 epochs, increased mean average precision from 53% (trained from scratch) to 81% while more than halving training time [24]. Clinically, Özbay et al. applied Mask R-CNN to 1050 periapical radiographs to detect fractured endodontic instruments, reporting mAP of 98.8% and an F1 score of 96.97%, surpassing YOLOv8 and matching specialist performance [25]. Kanwal et al. used Mask R-CNN on 1500 panoramic radiographs for tooth segmentation, achieving 98% accuracy, an F1 score of 88%, and 99% specificity, outperforming ten unsupervised baselines [26]. In 3-D terms, Cui et al. incorporated a cascaded 3-D Mask R-CNN into an AI system that segmented individual teeth and alveolar bone on 4938 CBCT scans with mean Dice coefficients of 91.5–94% and a throughput roughly 500-fold faster than manual annotation [27]. Collectively, these studies demonstrate Mask R-CNN’s versatility for dental detection, numbering, pathology mapping, and surgical planning, reinforcing its central role in contemporary computer-aided diagnostics.

Despite the close conceptual kinship between Faster R-CNN and Mask R-CNN, the literature lacks a systematic, head-to-head synthesis of their performance specifically for dental caries detection. Crucially, existing AI syntheses have not conducted a modality-stratified, head-to-head evaluation of Faster R-CNN versus Mask R-CNN (radiographs vs. photographs), which remains essential for translating model choice into practice. Most reports evaluate a single architecture or use heterogeneous metrics and datasets, preventing direct comparison and offering little guidance on when the instance-segmentation head in Mask R-CNN yields a tangible advantage over Faster R-CNN’s two-stage detector. This evidentiary gap is clinically consequential: selecting the right algorithm for a given imaging workflow can influence early lesion detection, minimally invasive treatment planning, and resource allocation in both screening and diagnostic settings.

Accordingly, this systematic review and meta-analysis (i) compares the diagnostic accuracy of Faster R-CNN and Mask R-CNN for caries detection, and (ii) performs modality-stratified analyses (radiographs versus photographs) and context-specific analyses (general screening vs. comprehensive diagnostic) of these CNN models. Our overarching clinical objective is to determine which algorithm performs better for each imaging modality and under which circumstances, thereby providing actionable recommendations for deployment in real-world dental care.

## 2. Methods

This systematic review and meta-analysis report was prepared in line with the Preferred Reporting Items for Systematic Reviews and Meta-Analyses of Diagnostic Test Accuracy Studies (PRISMA-DTA) guidelines [28]. The protocol was prospectively registered in the PROSPERO International Prospective Register of Systematic Reviews (CRD420251074443).

### 2.1. Literature Search

A comprehensive search was performed in PubMed (MEDLINE), Embase, Web of Science, and Scopus, with the final update on 15 June 2025. Search terms combined concepts for dental caries (e.g., “oral”, “dental”, “tooth disease”, “dental caries”, “dental cavities”) with deep-learning/detection keywords (e.g., “convolutional neural networks”, “Faster R-CNN”, “Mask R-CNN”). The full database-specific strategies are provided in Supplementary File S1. Searches were limited to English-language publications, with no restrictions on publication year, participant age, or country.

### 2.2. Inclusion Criteria

Studies were eligible if they:

1. Reported original diagnostic accuracy evaluations of Faster R-CNN and/or Mask R-CNN for dental caries detection using dental images (radiographic and/or photographic) compared against a reference standard (or clearly defined alternative verification).
2. Used cross-sectional or retrospective designs in which model outputs were evaluated against an established caries status.
3. Reported sufficient performance information to allow extraction of sensitivity/specificity and/or derivation of 2 × 2 data (TP/FP/TN/FN).
4. Included comparative evaluations against other automated approaches or human assessment, where applicable.

### 2.3. Exclusion Criteria

We excluded:

5. Non-original reports (e.g., case reports, commentaries, editorials, letters, narrative/systematic reviews) and conference abstracts, as well as animal experiments.
6. Studies lacking enough information to extract or compute diagnostic performance measures.
7. Studies not addressing caries detection (or not applying Faster R-CNN/Mask R-CNN as the index test of interest).

### 2.4. Study Selection and Data Extraction

All records were imported into EndNote^®^ v21 (Clarivate, Philadelphia, PA, USA) for de-duplication. Two authors (Quang Tuan Lam and Minh Huu Nhat Le) independently conducted the search and study selection. Titles and abstracts were screened to identify potentially eligible articles, followed by full-text assessment against the inclusion criteria by the same two authors. Reference lists of included studies were manually searched to identify additional records. Quang Tuan Lam extracted data using a standardized Microsoft Excel form; the form was pilot-tested on five included studies and approved by all of 4 authors, and then the extracted data were cross-checked by another author. Discrepancies were resolved through discussion or, when needed, consultation with co-authors (Nguyen Quoc Khanh Le, I-Ta Lee, and Minh Huu Nhat Le). Inter-reviewer agreement for title/abstract screening and full-text selection was assessed using Cohen’s kappa statistic (κ = 0.85); disagreements were resolved through discussion and consensus.

For general characteristics of included studies, we extracted data as authors/year, machine learning algorithms, number of annotators/qualifications, consensus approach, blinding, and caries definition. For performance metrics, we recorded study characteristics (first author, country, year); the evaluated algorithms (Faster R-CNN, Mask R-CNN); imaging modality (e.g., bitewing radiograph, periapical radiograph, intraoral photograph); dataset size and any train/validation/test splitting; reported performance metrics (accuracy, precision/PPV, sensitivity/recall, F1-score, specificity, AUC); and, where possible, 2 × 2 contingency data (TP, FP, TN, FN) for caries detection. TP/FP/TN/FN were extracted at the operating threshold applied in each study as reported by the authors. If a paper reported multiple thresholds, we prioritized the threshold used for the primary (main) analysis. Since this meta-analysis review used a bivariate random-effects model and the HSROC framework, differences in thresholds across studies are inherently accommodated in the pooled estimates and HSROC visualization.

### 2.5. Methodological Quality and Risk of Bias Assessment

Two authors (Quang Tuan Lam and Minh Huu Nhat Le) independently evaluated the methodological quality of each included study. Details regarding the imaging types, image resolution, equipment, camera settings, and standardization processes for each paper are provided in Supplementary Files S2 and S3. The risk of bias and applicability concerns were assessed using the Quality Assessment of Diagnostic Accuracy Studies—Artificial Intelligence (QUADAS-AI) tool [29], built on the QUADAS-2 framework [30] for AI-centered diagnostic accuracy studies. Two reviewers independently evaluated each included study across the QUADAS-AI domains—patient selection, index test (AI model), reference standard, and flow and timing—using signaling questions to guide judgments on risk of bias and applicability. For patient selection, we examined sampling strategy, clarity of inclusion/exclusion criteria, and whether image-quality-related exclusions could introduce selection/spectrum bias. For the index test, we assessed AI-specific risks such as transparency of train/validation/test splits, potential data leakage (e.g., patient-level overlap), prespecification of operating thresholds, and reporting of preprocessing/augmentation. For the reference standard, we evaluated the appropriateness and description of ground-truth labeling (criteria, annotator credentials, and consensus procedures) and blinding where relevant. For flow/timing, we assessed completeness of case inclusion, consistency of the reference standard, handling of missing/uninterpretable data, and any timing issues that could plausibly bias results. Each domain was rated as low, high, or unclear risk of bias, and applicability was rated as low, high, or unclear concern. Disagreements were resolved by discussion and, if needed, adjudicated by a third reviewer (Nguyen Quoc Khanh Le). The full results of Quality Assessment of Diagnostic Accuracy Studies-AI (QUADAS-AI) are provided in Supplementary Table S1.

For studies utilizing radiographic images and radiomics workflows, we additionally assessed methodological quality and reporting rigor using the Radiomics Quality Score (RQS) proposed by Lambin et al. [31]. RQS consists of 16 items covering key aspects of radiomics study design, analysis, and validation. Specifically, we evaluated: (i) image acquisition and protocol reporting (including whether acquisition parameters were sufficiently described and whether robustness/reproducibility was assessed across scanners or settings); (ii) segmentation procedures and feature robustness (e.g., use of multiple segmentations/readers, assessment of inter-/intra-observer variability, and stability of extracted features); (iii) feature engineering and statistical control (appropriate feature reduction/selection, correction for multiple testing, and avoidance of overfitting); (iv) model development and validation (clear train/validation/test strategy, internal validation such as cross-validation/bootstrapping, and external validation on independent cohorts where available); (v) clinical utility and comparison to relevant baselines (e.g., comparison to clinician performance or standard clinical models, and decision-curve analysis where applicable); and (vi) transparency and evidence level (e.g., availability of code/data or sufficient methodological detail for reproducibility, and higher-level evidence such as prospective or multi-center design). Each item was scored according to the published RQS rubric to yield a total score ranging from 0 to 36, with higher scores indicating better methodological rigor and reproducibility. RQS scoring was performed independently by the same two reviewers, and discrepancies were resolved by consensus or, when necessary, adjudication by the third reviewer (Nguyen Quoc Khanh Le). The final assessment of Radiomics Quality Score (RQS) can be found in Supplementary Table S2.

### 2.6. Data Synthesis and Analysis

The primary aim of this systematic review and meta-analysis was to quantify the overall diagnostic accuracy of Faster R-CNN and Mask R-CNN for dental caries detection using standard performance measures, and the secondary aim was to examine whether study- or image-level characteristics influenced model performance. Because Faster R-CNN and Mask R-CNN were evaluated across independent study cohorts rather than within-study head-to-head designs, all between-model comparisons were treated as indirect. We synthesized extracted diagnostic accuracy data using a bivariate random-effects (Reitsma) model to generate pooled estimates of sensitivity and specificity with corresponding 95% confidence intervals (CIs), accounting for both within-study sampling error and between-study variability. To illustrate the sensitivity–specificity trade-off, we constructed a summary ROC (sROC) curve within a hierarchical diagnostic accuracy framework. Between-study heterogeneity was summarized by reporting τ^2^ for logit-transformed sensitivity and specificity; for interpretability, we additionally fitted univariate random effects models for sensitivity and specificity and reported I^2^ for each outcome. To explore potential sources of heterogeneity and adjust for major study-level confounders, prespecified meta-regression and stratified analyses were conducted within the same hierarchical framework to evaluate the impact of covariates on pooled sensitivity, specificity, and AUC. Covariates included imaging modality (radiographic vs. photographic), dataset size, presence of external validation, and study design (retrospective vs. prospective). Subgroup analyses were additionally performed by imaging modality. All analyses were conducted in R (version 4.4.2; R Foundation for Statistical Computing, Vienna, Austria), and statistical significance was defined as *p* < 0.05.

## 3. Results

### 3.1. Study Selection

**Figure 3:**
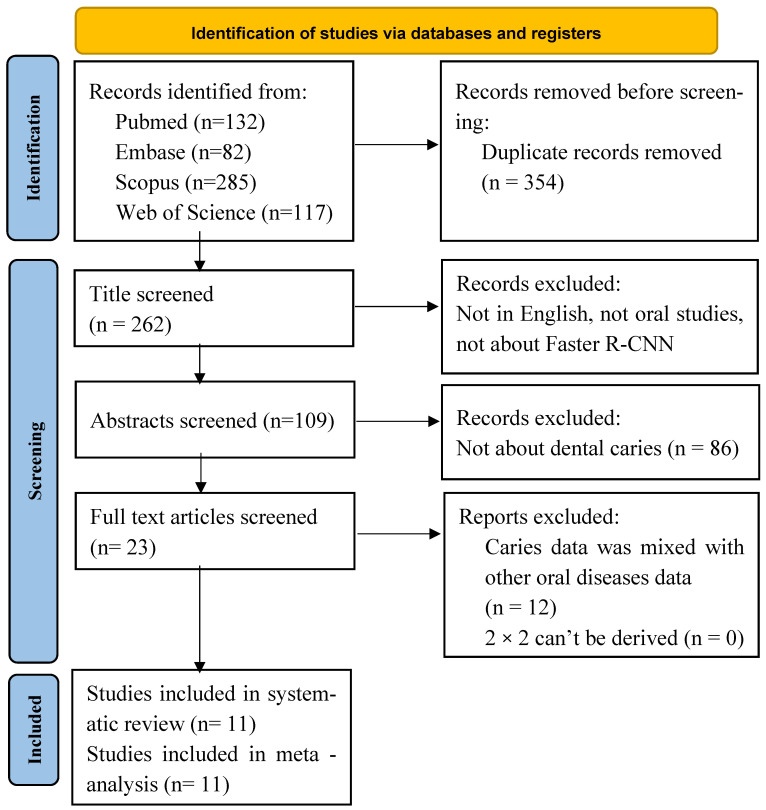
PRISMA-DTA flow diagram summarizing the identification, screening, eligibility assessment, and inclusion of studies evaluating Faster R-CNN for caries detection.

Faster R-CNN: The search identified 616 records. After removing duplicates, 354 unique records remained for title/abstract screening. We assessed 23 full texts for eligibility, of which 11 studies [32,33,34,35,36,37,38,39,40,41,42] met the inclusion criteria and were included in the qualitative synthesis; all 11 provided sufficient data for meta-analysis (Figure 3).

**Figure 4:**
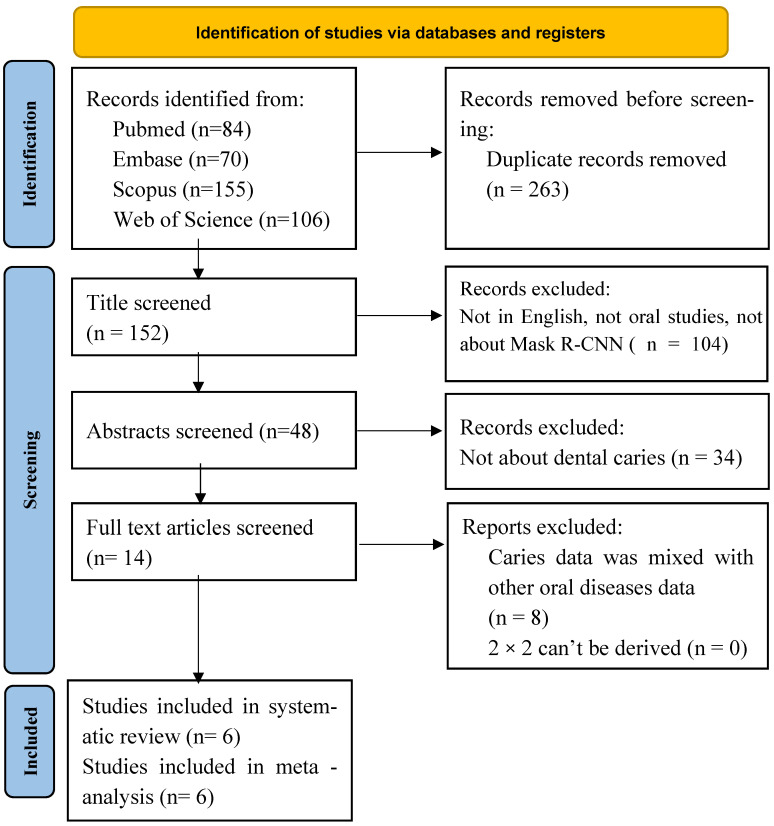
PRISMA-DTA flow diagram summarizing the identification, screening, eligibility assessment, and inclusion of studies evaluating Mask R-CNN for caries detection.

Mask R-CNN: A similar screening and eligibility process was applied. Six studies [43,44,45,46,47,48] satisfied the inclusion criteria and were included in both the qualitative synthesis and the meta-analysis (Figure 4).

### 3.2. Study Characteristics

Sixteen of the 17 included studies were published from 2022 onward and originated from researchers in nearly 20 countries, reflecting increasing global interest in these DL models. Characteristics of included studies such as authors/year, machine learning algorithms, number of annotators/qualifications, consensus approach, blinding and caries definition can be found in Table 1. Across studies, a total of 41,384 tooth images were analyzed (after augmentation), with sample sizes ranging from 90 to 12,750 images per study. Faster R-CNN was used in 11/17 studies (64.7%), while Mask R-CNN was evaluated in the remaining 6/17. Radiographic images were used in 10/17 studies (58.8%) and intraoral photographs in 8/17 (47.1%), with Rashid et al. [48] including both modalities. Data augmentation was reported in all studies to expand and/or balance training data. Only 6 studies (35.3%) incorporated an explicit segmentation step, consistent with the Mask R-CNN workflow. Overall, the included studies aimed to develop AI-based tools to improve the accuracy and efficiency of caries detection in clinical settings.

### 3.3. Methodological Quality and Risk of Bias of Included Studies

**Figure 5:**
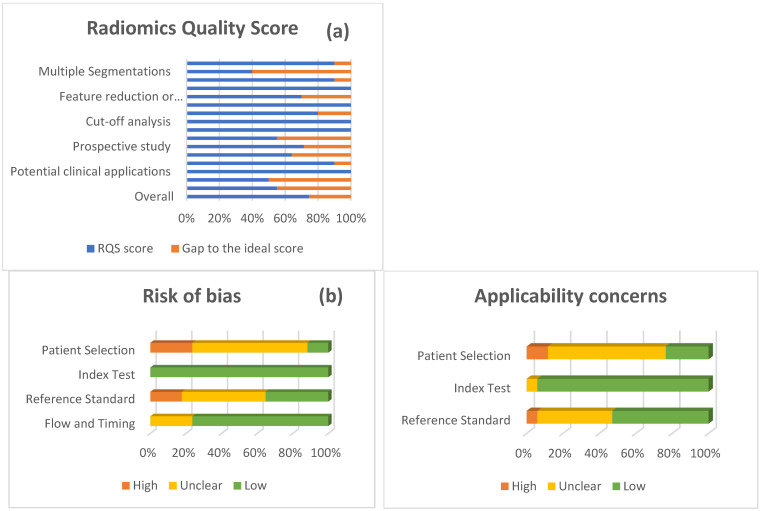
Quality assessment of included studies using the Radiomics Quality Score (RQS) and QUADAS-AI. (**a**) Mean RQS across included radiomics studies, shown as the percentage of the maximum score by domain. (**b**) Risk of bias and applicability concerns summary: distribution of reviewers’ QUADAS-AI judgments for each item, presented as percentages across all included studies (*n* = 17). QUADAS-AI = Quality Assessment of Diagnostic Accuracy Studies–Artificial Intelligence.

Overall, the methodological quality of the included studies was of a fairly good standard. According the Figure 5, the mean RQS for studies involving radiographic images was 74.4% (268/360) of the maximum score (range: 55.6–91.7%), indicating generally well-structured radiomics study design and reporting while still leaving room for improvement. The lowest RQS was reported in Velusamy et al., (2024) (20/36; 55.6%) [41], whereas the highest score was observed in E. Chaves et al., (2024) (33/36; 91.7%) [43]. This variability in RQS suggests meaningful differences in study rigor that may partly contribute to the observed between-study heterogeneity. The most common shortcomings were the lack of multiple segmentations, omission of calibration statistics, absence of prospective design or robust validation, and failure to conduct cost-effectiveness analyses or provide open data. In contrast, nearly all studies reported basic discrimination metrics (e.g., AUC or accuracy), compared model outputs against an appropriate ground-truth “gold standard,” and discussed potential clinical applications.

Using QUADAS-AI, we found generally low concern for applicability across studies, although several risks of bias were identified. For patient selection, four studies were judged to have a high risk of bias, most commonly because images or patients were not selected randomly (e.g., retrospective sampling or exclusion of certain cases), which may introduce selection bias. The index test domain was rated as low risk of bias in all studies, as caries detection was performed using an objective algorithm without knowledge of the reference standard outcomes. Likewise, the flow and timing domain was low risk in most studies (13/17), since the index test and reference standard were typically applied to the same images (making the time interval not applicable) and/or all cases received reference standard verification of caries status. By contrast, the reference standard domain was unclear in approximately half of the studies (47.1%), largely because papers did not consistently report whether reference assessments (e.g., clinical examination or expert radiographic reading) were blinded to the algorithm results or how rigorously the reference diagnosis was established.

Regarding applicability, patient selection raised the greatest concern, with 76.5% of studies classified as high/unclear concern. A similar pattern was observed for the reference standard, where 9 of 17 studies were assessed as low concern. In contrast, the index test domain showed the most favorable applicability profile, with no studies rated as having high concern.

### 3.4. Quantitative Analysis (Meta-Analysis)

**Figure 6:**
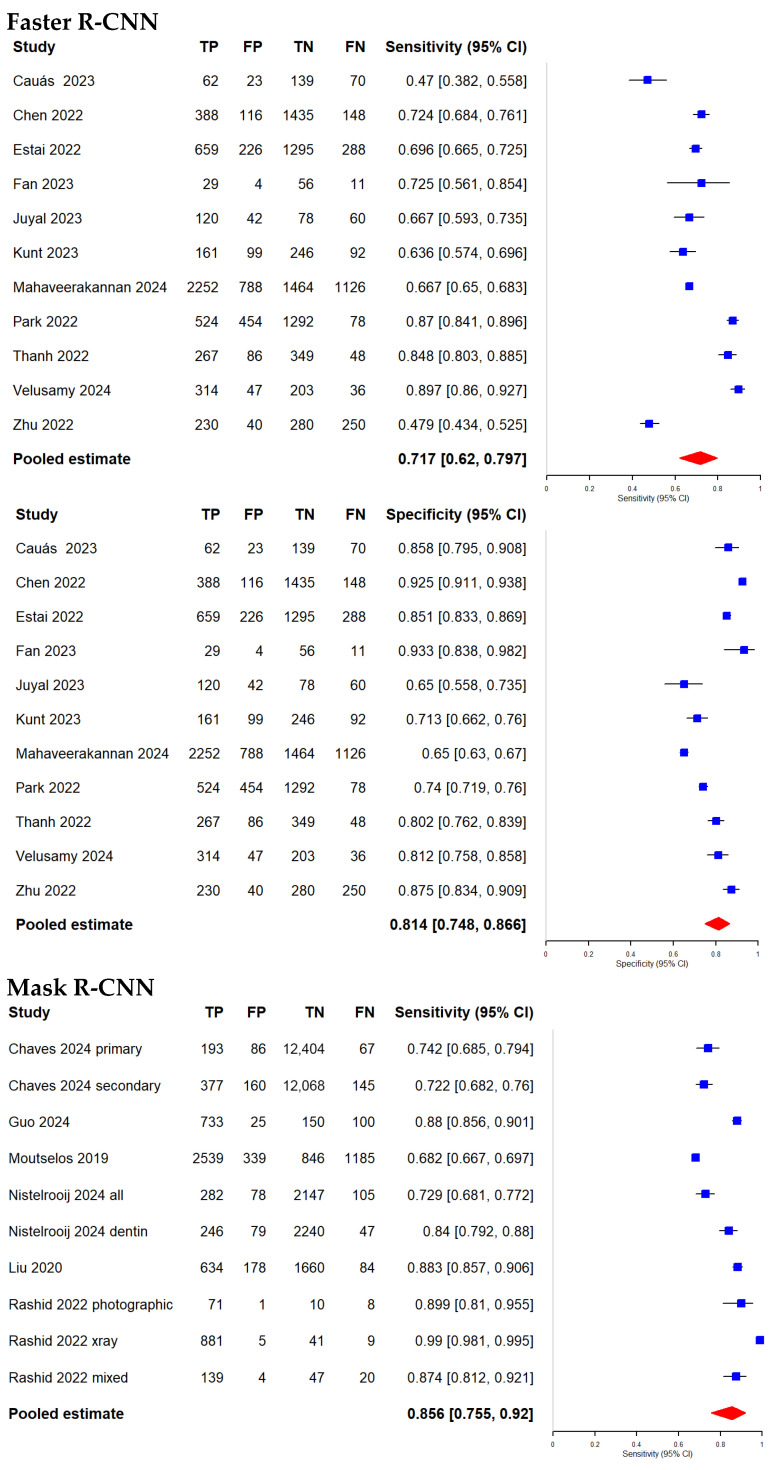

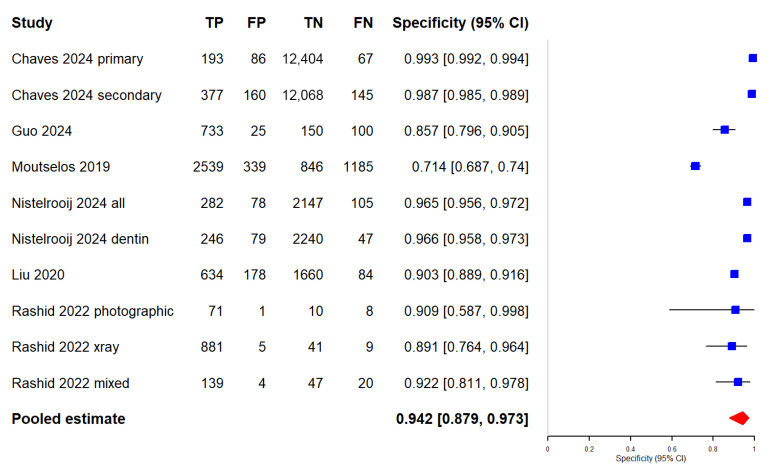
Forest plots of studies evaluating Faster R-CNN (k = 11) [32,33,34,35,36,37,38,39,40,41,42] and Mask R-CNN (k = 6) [43,44,45,46,47,48] for dental caries detection.

**Figure 7:**
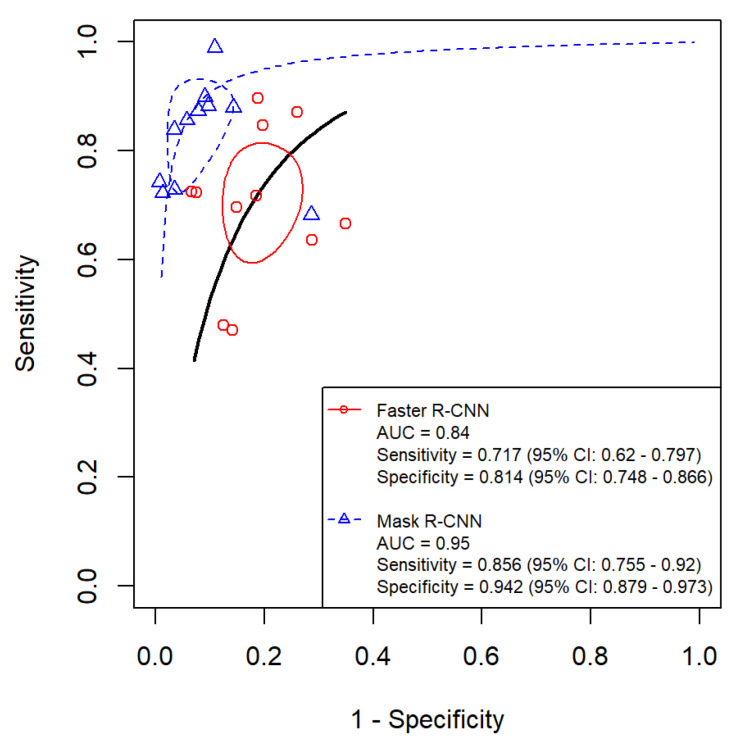
HSROC curves comparing Faster R-CNN and Mask R-CNN for dental caries detection across all included studies.

**Figure 8:**
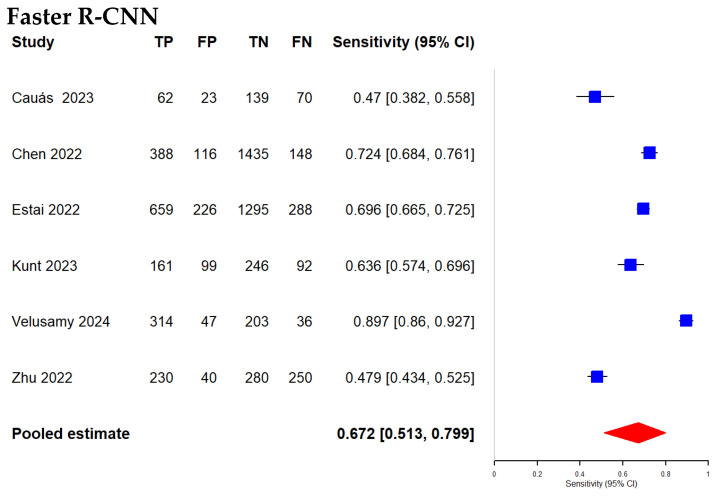

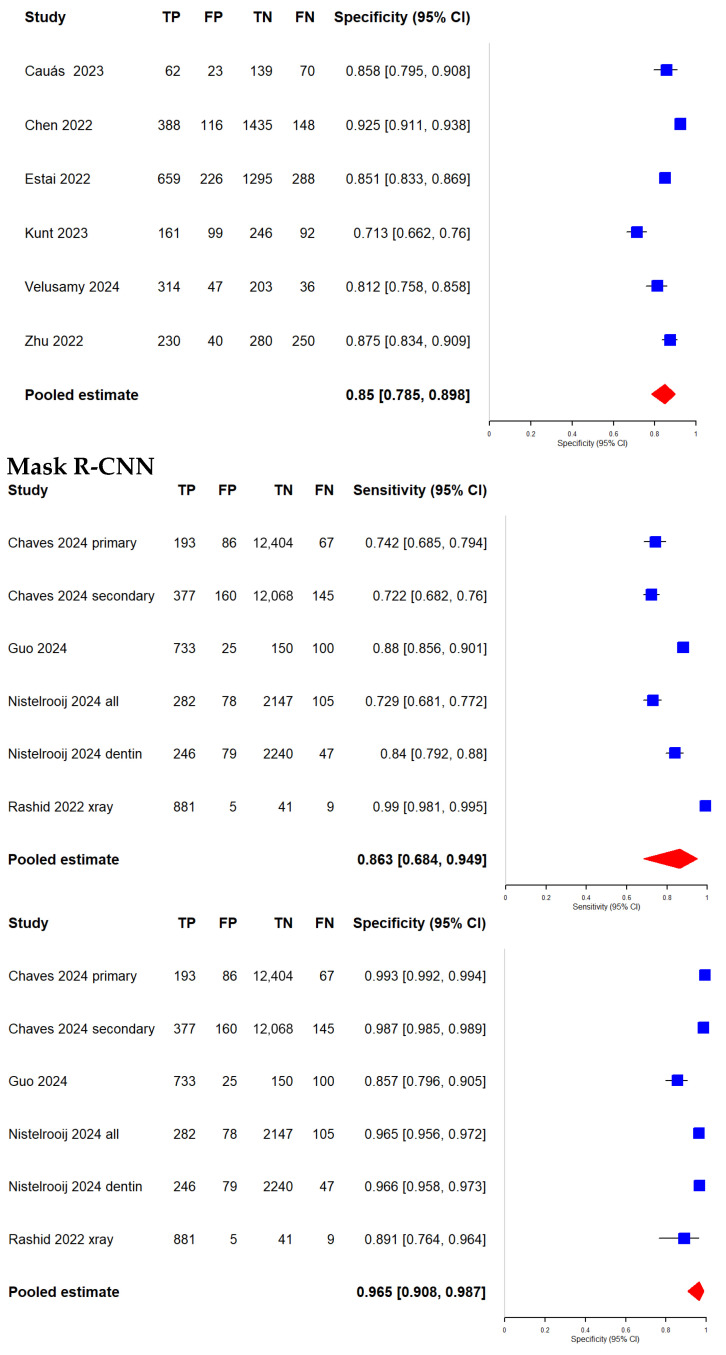
Forest plots of radiographic-image studies evaluating Faster R-CNN (k = 6) [32,33,34,37,41,42] and Mask R-CNN (k = 4) [43,44,46,48] for dental caries detection.

**Figure 9:**
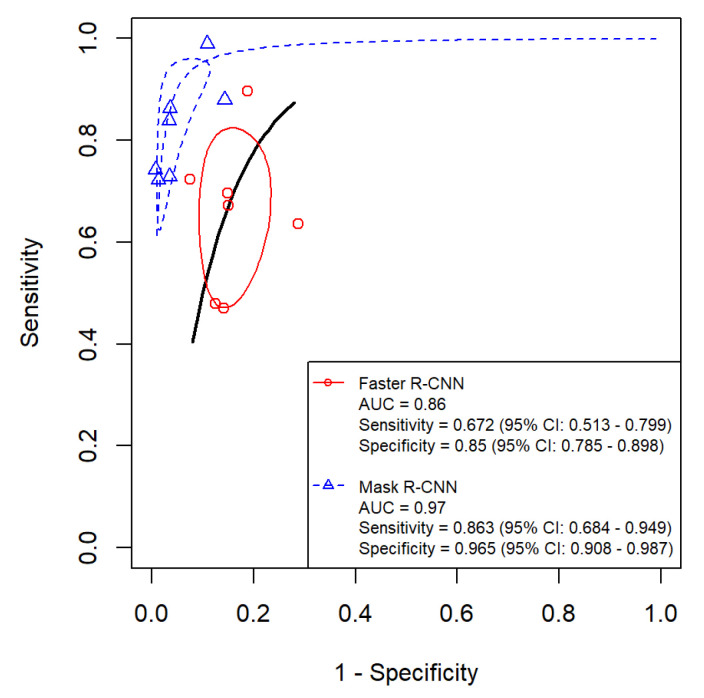
HSROC curves comparing Faster R-CNN and Mask R-CNN for dental caries detection using radiographic images.

**Figure 10:**
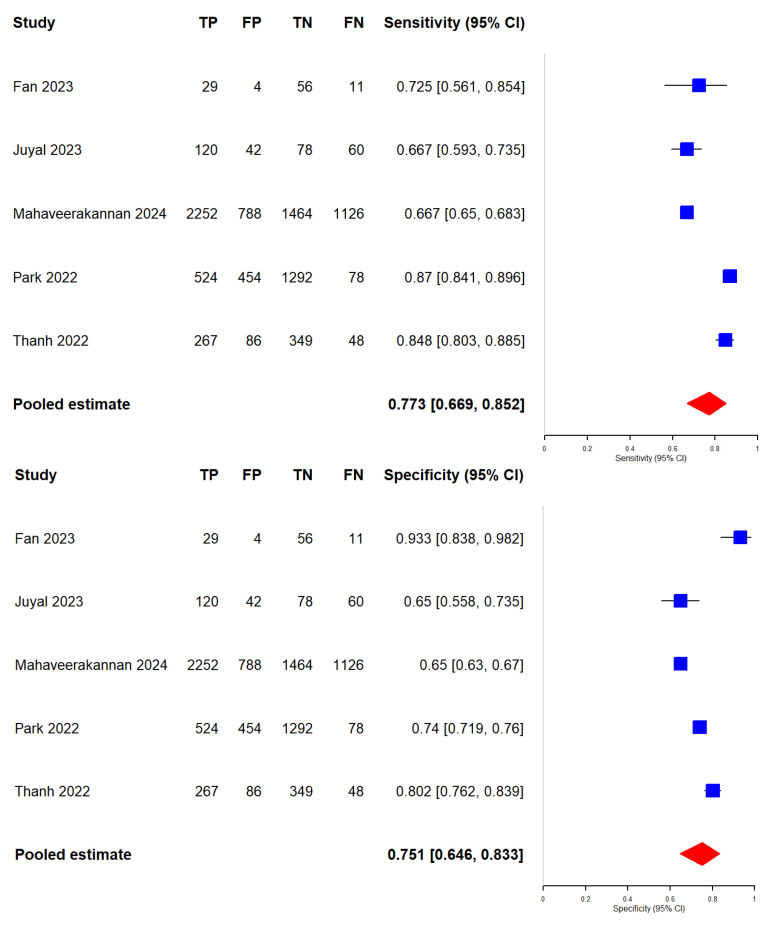

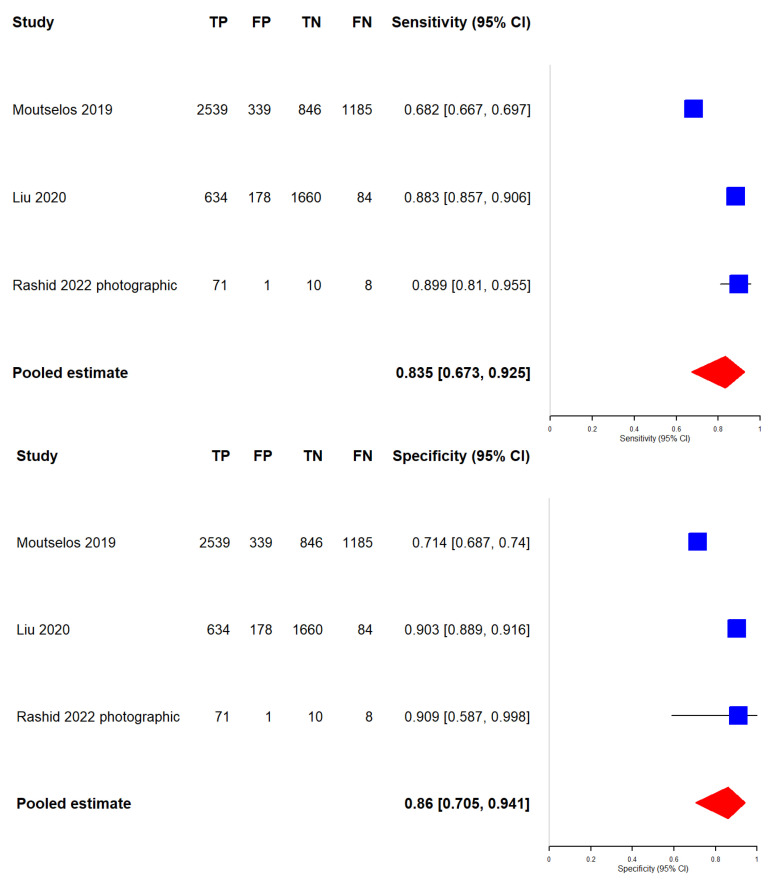
Forest plots of photographic image studies evaluating Faster R-CNN (k = 5) [35,36,38,39,40] and Mask R-CNN (k = 3) [45,47,48] for dental caries detection.

**Figure 11:**
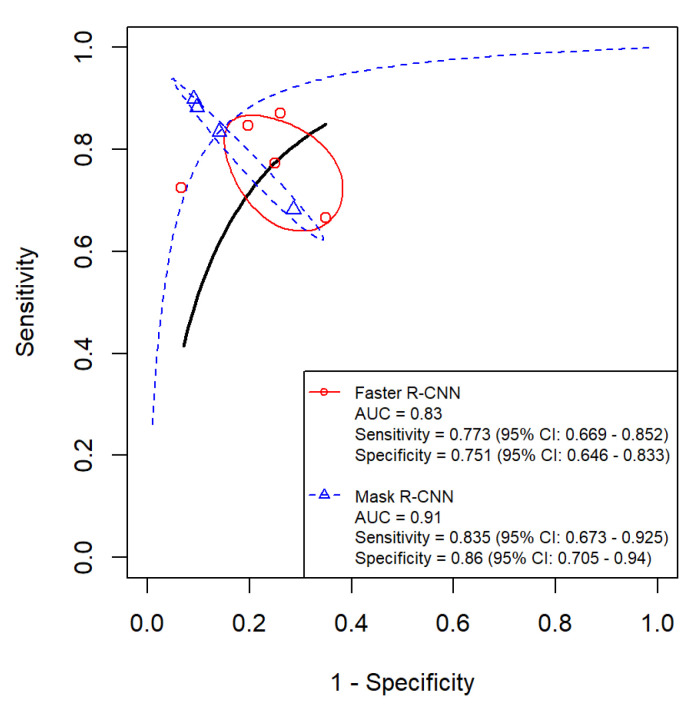
HSROC curves comparing Faster R-CNN and Mask R-CNN for dental caries detection using photographic images.

We included 11 studies [32,33,34,35,36,37,38,39,40,41,42] and 6 studies [43,44,45,46,47,48] in our meta-analysis evaluating the diagnostic accuracy of the Faster R-CNN and Mask R-CNN algorithms, respectively, for detecting dental caries. Performance metrics of Faster R-CNN papers and Mask R-CNN papers can be found in Table 2 and Table 3, respectively. Additionally, we categorized the included studies based on the type of dental images used—radiographic or photographic—to examine potential differences in performance between the two algorithms across these imaging modalities. Importantly, all between-algorithm contrasts are indirect because the Faster R-CNN and Mask R-CNN estimates come from different sets of studies, and no head-to-head evaluations on the same datasets under identical annotation protocols and operating thresholds were available. Between-study heterogeneity was consistently high (I^2^ > 90% for most outcomes), with larger between-study variance for specificity in Mask R-CNN overall (τ^2^ = 2.577), while modality-specific analyses reduced—but did not eliminate—heterogeneity (radiograph τ^2^_Specificity = 0.885; photograph τ^2^_Specificity = 0.856). The results of the meta-analysis are presented in Figure 6, Figure 7, Figure 8, Figure 9, Figure 10 and Figure 11 and summarized in Table 4.

### 3.5. Evaluation of the Diagnostic Accuracy of Faster R-CNN and Mask R-CNN Algorithms

We generated forest plots (Figure 6) and hierarchical sROC curves (Figure 7) to summarize study-level and pooled diagnostic performance for both algorithms. Pooled estimates are reported with 95% CIs. Given the high heterogeneity across studies, pooled estimates and indirect contrasts should be interpreted cautiously, since observed differences may reflect study-level factors such as dataset composition, lesion definitions, annotation procedures, and threshold selection rather than algorithm effects alone.

Sensitivity: Faster R-CNN achieved a pooled sensitivity of 71.7% (62.0–79.7%), with study-level estimates ranging from 47.0% to 89.7%. Mask R-CNN yielded a pooled sensitivity of 85.6% (75.5–92.0%), with a range of 68.2% to 99.0%. The indirect Z-test for proportions suggested a statistical difference between pooled sensitivities (*p* = 0.0244); however, this reflects an indirect contrast across non-overlapping study sets and should be interpreted in the context of very high between-study heterogeneity.

Specificity: Faster R-CNN showed a pooled specificity of 81.4% (74.8–86.6%), with study-level estimates ranging from 65.0% to 93.3%. Mask R-CNN achieved a pooled specificity of 94.2% (87.9–97.3%), with a range of 71.4% to 99.3%. The indirect Z-test suggested a statistical difference between pooled specificities (*p* = 0.00089), but this finding remains subject to the same limitations of indirect comparison and substantial heterogeneity.

AUC: Mask R-CNN had a higher pooled AUC than Faster R-CNN (0.95 vs. 0.84). DeLong’s test suggested a statistical difference (*p* = 0.0053). This AUC contrast is also indirect and should be interpreted cautiously given the high residual heterogeneity.

### 3.6. Comparison of Diagnostic Accuracy of Faster R-CNN and Mask R-CNN Algorithms with Radiographic Images

In radiographic studies, Faster R-CNN had a pooled sensitivity of 67.2% (95% CI: 51.3–79.9%), with study-level estimates ranging from 47.0% to 89.7%. Mask R-CNN had a pooled sensitivity of 86.3% (95% CI: 68.4–94.9%), with estimates ranging from 72.2% to 99.0%. The indirect Z-test suggested a statistical difference (*p* = 0.0497). This contrast remains indirect, since the two algorithms were assessed in different study sets, and heterogeneity persisted despite modality stratification.

For specificity, Faster R-CNN yielded a pooled estimate of 85.0% (95% CI: 78.5–89.8%) with a range of 71.3% to 92.5%. Mask R-CNN yielded 96.5% (95% CI: 90.8–98.7%) with a range of 85.7% to 99.3%. The indirect Z-test suggested a statistical difference (*p* = 0.00105), and interpretation should remain cautious because residual heterogeneity was not eliminated.

For overall discrimination, Mask R-CNN showed a higher pooled AUC than Faster R-CNN (0.97 vs. 0.86). DeLong’s test suggested a statistical difference (*p* = 0.0067). This result reflects an indirect contrast and may be influenced by study-level differences across radiographic datasets and evaluation thresholds.

### 3.7. Comparison of Diagnostic Accuracy of Faster R-CNN and Mask R-CNN Algorithms with Photographic Images

In photographic studies, Faster R-CNN showed a pooled sensitivity of 77.3% (95% CI: 66.9–85.2%), with study-level estimates ranging from 66.7% to 87.0%. Mask R-CNN showed a pooled sensitivity of 83.5% (95% CI: 67.3–92.5%), with estimates ranging from 68.2% to 89.9%. The indirect Z-test did not indicate a statistical difference (*p* = 0.435).

For specificity, Faster R-CNN yielded a pooled estimate of 75.1% (95% CI: 64.6–83.3%) with a range of 65.0% to 93.3%, while Mask R-CNN yielded 86.0% (95% CI: 70.5–94.1%) with a range of 71.4% to 90.9%. The indirect Z-test did not indicate a statistical difference (*p* = 0.156).

For overall discrimination, Mask R-CNN had a higher pooled AUC than Faster R-CNN (0.91 vs. 0.83). DeLong’s test suggested a statistical difference (*p* = 0.048). This AUC result should be interpreted cautiously because it is an indirect contrast, it is near the conventional significance threshold, sensitivity and specificity differences were not statistically significant, and residual heterogeneity remained. Therefore, this finding may not translate into clinically meaningful improvement without prospective head-to-head evaluations that apply standardized annotation protocols and uniform operating thresholds within the same photographic datasets.

### 3.8. Meta-Regression and Subgroup Analysis

Table 4 presents the results of subgroup analysis; in this analysis, we compared the effects of different covariates on summary estimates.

## 4. Discussion

Dental caries (tooth decay) remains one of the most common oral diseases globally and a major contributor to the worldwide burden of oral conditions [49]. Its development is multifactorial, but a key driver is frequent consumption of free sugars, which fuels acid production by dental plaque biofilms and is consistently associated with a higher risk of caries [50]. Caries and other oral diseases are also strongly socially patterned—poverty and constrained access to care amplify risk and untreated disease—while the economic burden is substantial, with estimates that direct treatment costs for dental diseases accounted for nearly 5% of global health expenditure [49]. Because lesions can progress and ultimately compromise tooth structure and function, timely identification of lesion location/severity is essential for appropriate preventive or minimally invasive management, particularly in settings with high baseline risk and limited resources [51].

Dental informatics applies computer and information sciences to dentistry to support and augment clinical workflows, including diagnostic decision-making [52]. Automated systems for detecting and classifying dental pathology can enable earlier diagnosis, reduce dependence on time-consuming manual assessment, and alleviate clinician workload, thereby improving oral health and preventing complications [53]. While machine-learning methods have long been applied to medical imaging, deep learning (DL) has gained prominence because these models can learn hierarchical feature representations directly from raw images, often improving performance in tasks such as detection, segmentation, and classification [54]. Convolutional neural networks (CNNs)—a core class of deep learning models—are now widely used for medical and dental computer vision tasks (e.g., classification, detection, and segmentation), and have become a methodology of choice in medical image analysis [54]. In dental radiology, CNN-based CAD systems are increasingly discussed as workflow support tools that can help clinicians interpret images more efficiently and consistently, with the potential to reduce diagnostic workload and human error when used as an adjunct to expert review [55]. Among DL detectors, Faster R-CNN and Mask R-CNN are prominent for object detection and segmentation. Faster R-CNN is a two-stage framework that first proposes regions and then refines them for accurate bounding box predictions [15]. Mask R-CNN extends this architecture with a mask branch for pixel-level instance segmentation, enabling simultaneous detection, classification, and mask generation [22]. Both architectures use a shared convolutional backbone to extract feature maps and leverage region-based representations to capture object appearance and spatial context for localization and segmentation.

Following database searching, title/abstract screening, and full-text assessment, 11 Faster R-CNN studies and 6 Mask R-CNN studies were included in the systematic review; all 17 studies contributed data to the meta-analysis. All papers were published in 2022 or later and represent contributions from multiple countries, underscoring the growing global interest in applying Faster R-CNN and Mask R-CNN to caries detection. Across studies, the shared aim was to develop AI-enabled tools to enhance diagnostic accuracy in clinical practice.

Regarding RQS, included radiographic studies performed well on criteria such as basic discrimination statistics, comparison with a “gold standard,” and articulation of potential clinical applications (>95%). However, multiple segmentations, calibration statistics, prospective design, and cost-effectiveness analyses were infrequently addressed. The mean RQS was 74.4% (range 55.6–91.7%). QUADAS-AI results were likewise encouraging: the “index test” domain generally showed low risk of bias and low applicability concerns, as did “flow and timing.” The least favorable ratings appeared in “patient selection,” followed by “reference standard.” This pattern likely reflects reliance on retrospectively sampled public datasets without clear reporting of consecutive or random inclusion, limiting assessment of selection bias. In addition, ground-truth labeling procedures were often insufficiently documented (e.g., single- vs. multi-expert annotation), raising questions about the adequacy and independence of the reference standard. Heterogeneity may also arise from differences in design (retrospective vs. prospective) and imaging modality. For example, bitewing radiograph studies showed lower heterogeneity in specificity (narrower CIs in subgroup analyses), consistent with standardized acquisition, whereas photographic studies exhibited greater variability, potentially driven by lighting inconsistency and annotation subjectivity [56]. Overall, RQS and QUADAS-AI profiles indicate generally well-structured designs and reporting while highlighting the need for modality-tailored guidelines for DL applications in oral health.

Both Faster R-CNN and Mask R-CNN achieved satisfactory diagnostic performance, but Mask R-CNN was superior across metrics, with higher pooled sensitivity (85.6% vs. 71.7%, *p* = 0.0244), specificity (94.2% vs. 81.4%, *p* = 0.00089), and AUC (0.95 vs. 0.84, *p* = 0.0053). These gains are consistent with several architectural advantages. Mask R-CNN preserves the two-stage proposal pipeline of Faster R-CNN while adding a mask head that provides pixel-level supervision, enabling true instance segmentation—crucial for delineating subtle enamel–dentin borders. RoIAlign further preserves spatial precision relative to RoIPooling, avoiding rounding artifacts and aligning with the higher pooled sensitivity observed for Mask R-CNN. In addition, backbones are typically paired with a Feature Pyramid Network, granting multiscale representation that improves detection of small, low-contrast cavities commonly seen on bitewings [24]. Beyond caries, results from IEEE BIBM 2018 suggest the Mask R-CNN framework transfers effectively to other oral mucosal lesions, indicating versatility for subtle dental imaging tasks [57]. Collectively, precise alignment, multiscale feature fusion, and mask-level supervision likely underpin the statistically significant advantage of Mask R-CNN, positioning it as a robust option for automated screening pipelines.

Modality-specific analyses revealed important differences. For radiographic images, Mask R-CNN outperformed Faster R-CNN in pooled sensitivity (86.3% vs. 67.2%, *p* = 0.0497), specificity (96.5% vs. 85.0%, *p* = 0.00105), and AUC (0.97 vs. 0.86, *p* = 0.0067), mirroring the overall results. In photographic images, Mask R-CNN again showed higher pooled sensitivity (83.5% vs. 77.3%) and specificity (86.0% vs. 75.1%), but these differences were not statistically significant (*p* = 0.435 and 0.156, respectively); AUC favored Mask R-CNN (0.91 vs. 0.83, *p* = 0.048). These findings suggest Mask R-CNN is particularly well suited to radiographic workflows, where its mask branch can disentangle overlapping anatomic shadows intrinsic to bitewings and other radiographs. RoIAlign and FPN help resolve faint radiolucent margins indicating early dentinal demineralization—features that a box-only pipeline may blur. By contrast, photographic images often contain strong color/texture cues that allow bounding box detectors to localize lesions adequately; segmentation adds less incremental information and may be hindered by glare, saliva, and depth-of-field artifacts that disrupt mask continuity, yielding non-significant sensitivity–specificity margins [56]. Moreover, the photographic subgroup results likely reflect substantial heterogeneity in image acquisition and labeling. Unlike radiographs, intraoral photographs are frequently captured under non-standardized conditions, with large variability in image quality driven by differing lighting (illumination intensity, color temperature, shadows), camera angle, focus/depth-of-field, motion blur, and saliva. These factors can obscure lesion boundaries and introduce inconsistent visual cues, further disrupting pixel-level mask continuity and increasing between-study variability. Furthermore, many photographic datasets are relatively small (<3000 frames) and annotated at coarse tooth-level granularity, limiting generalization for mask heads, whereas radiographic corpora more often provide dense, accurate contours that improve training [58]. Accordingly, the additional mask loss may act as an effective regularizer only when ground-truth masks are reliable; this is more typical in calibrated X-ray imagery than in variably illuminated intraoral photos, where boundaries can be visually unstable and labels relatively coarse. Consistent with this, He et al. reported that mask heads confer the greatest benefit when spatial precision is paramount [22]. Taken together, the integration of rich grayscale structural details, dense annotations, and inherent overlap artifacts renders radiographs particularly well-suited for Mask R-CNN, whereas photographic workflows yield only marginal improvements over Faster R-CNN. Importantly, comparisons between Faster R-CNN and Mask R-CNN in this meta-analysis are indirect because the two architectures were not evaluated head-to-head on the same datasets under identical annotation protocols and operating thresholds. Accordingly, differences in pooled performance may be driven by study-level heterogeneity, including imaging modality, dataset composition, lesion definition, annotation strategy, validation design, and threshold selection, rather than architecture alone. To mitigate this, we performed modality-stratified analyses and prespecified meta-regression adjusting for these covariates; however, residual confounding is likely. Definitive conclusions regarding architectural superiority require within-study head-to-head evaluations under identical experimental settings, which were not available in the current literature. In addition, very high heterogeneity in this meta-analysis means pooled sensitivity, specificity, and AUC should be interpreted as context specific summaries rather than universal benchmarks. Accuracy differed across studies due to variation in modality, acquisition, lesion definitions, annotation and reference standards, operating thresholds, and validation design. Thus, pooled estimates reflect an average across heterogeneous research settings and may not predict performance in a given clinic or deployment workflow. Consequently, for implementation, the most informative results are setting matched analyses, such as modality specific subgroups and externally validated cohorts, and future work should report prediction intervals to quantify expected performance dispersion across new populations and institutions.

Among radiographic modalities, bitewings were most frequently employed, consistent with prior literature. Bitewing radiographs are often used as a reference standard in clinical caries diagnosis [59] and are recommended for detecting early-stage and interproximal caries given their high sensitivity, low radiation dose, low cost, and rapid acquisition [60]. Consequently, radiograph-based caries detection can help prevent disease progression and reduce invasive treatments, while facilitating personalized care in high-risk patients [61]. Nonetheless, dental radiographs should be used judiciously due to ionizing radiation exposure, even though risks are generally low for most individuals [62]. Photographic imaging, by contrast, is radiation-free and highly accessible, including in rural settings with limited dental infrastructure. The ubiquity and affordability of smartphones in many countries support smartphone-based diagnostic tools that could expand screening reach. Indeed, recent biomedical research has leveraged mobile device sensors to deliver cost-effective solutions for remote dental care [63].

This publication should be interpreted alongside our prior YOLO-focused systematic review and meta-analysis on dental caries detection, which synthesized evidence for one-stage detectors tailored to rapid, real-time screening, where low latency, deployment simplicity, and coarse lesion localization often determine clinical feasibility (for example, chairside or mobile screening). By contrast, the present study was intentionally conducted as a separate analysis because Faster R-CNN and Mask R-CNN belong to a distinct family of two-stage, region-based detectors, and Mask R-CNN further adds an instance-segmentation head that can materially change localization precision, annotation burden, and error modes relative to one-stage box prediction. These architectural differences introduce model-specific heterogeneity (for example, region proposal behavior, reliance on RoIAlign, and sensitivity to mask supervision quality) that would be obscured if two-stage detectors were pooled with one-stage YOLO studies. Consistent with this tradeoff, YOLO can process dental radiographs in about 15 milliseconds on a mid-range GPU [64], whereas two-stage approaches typically require about 90 milliseconds, with runtimes extending to 270 milliseconds when deeper backbones are used or when CBCT volumes are processed [65]. An our meta-analysis encompassing 14 studies reported that YOLO achieved a pooled sensitivity of 79%, specificity of 85%, and an area under the ROC curve (AUC) of 0.83 for caries-level detection, indicating that its rapid processing is accompanied by moderate diagnostic performance [66], which remains significantly lower than that of Faster R-CNN and Mask R-CNN observed in the present study. Accordingly, the distinct technical contribution of this study is modality-stratified performance characterization for two-stage detectors and an indirect comparative synthesis of Faster R-CNN versus Mask R-CNN within a diagnostic accuracy meta-analytic framework, while explicitly recognizing that statistical differences across pooled study sets are not head-to-head evidence and may be confounded by study-level factors unless within-study comparisons are available. Clinically, these results inform when two-stage detection and, when appropriate, instance segmentation is more defensible for workflows requiring higher spatial precision and boundary delineation on radiographs (for example, subtle radiolucent lesions, interproximal or deeper disease, and longitudinal monitoring around restorations), while YOLO-based systems remain appropriate for fast detection-only screening in low-resource settings.

To the best of our knowledge, this is the first systematic review and meta-analysis to synthesize the diagnostic accuracy of Faster R-CNN and Mask R-CNN for dental caries detection, supporting earlier detection across diverse clinical settings. Our strengths include the inclusion of commonly used imaging modalities for caries assessment, enabling modality-specific comparisons across different technical and clinical settings. We also explained results based on technical aspects of not only two mentioned algorithms but also DL structures in general for a more comprehensive overview. However, several limitations should be noted, including the predominance of retrospective studies and substantial heterogeneity in patient characteristics and imaging parameters. Given the substantial between-study variability (high I^2^/τ^2^), we interpreted the pooled estimates cautiously, emphasizing that they provide only an overall summary and may not represent any single clinical setting. We therefore focused on the HSROC curve and reported subgroup-specific results rather than relying solely on a single pooled estimate. Additionally, we observed limited methodological rigor (suboptimal RQS and QUADAS-AI), limited reproducibility, potential publication bias, and insufficient standardization of imaging and labeling protocols. Lastly, no included study directly compared Faster R-CNN and Mask R-CNN on the same dataset under identical experimental conditions, precluding true head-to-head subset analysis and limiting causal attribution of performance differences to model architecture alone. These limitations should be explicitly acknowledged and addressed in future research to enhance methodological rigor and the reliability of evidence.

Future directions may further elevate performance through architectural, data, and deployment strategies. Beyond model design, future studies should strengthen methodological rigor through standardized acquisition/reporting for photographic and radiographic data, harmonized annotation (clear lesion definitions and labeling granularity, double-reading/consensus, and reporting inter-/intra-rater reliability), and prospective or consecutively sampled multi-center cohorts with prespecified analysis plans and independent external validation. Because the number of eligible studies on Faster R-CNN (k = 11) and Mask R-CNN (k = 6) remains limited, excluding studies with unclear reporting may further reduce statistical power. Nevertheless, future work should conduct sensitivity analyses that exclude studies with unclear or high risk of bias in the reference standard and/or patient selection domains and report the impact on pooled estimates. Moreover, because QUADAS-AI highlighted patient selection as the weakest domain, future studies should consider an inclusion preference by performing sensitivity analyses restricted to studies at low risk of bias for patient selection. Authors should also explicitly discuss spectrum bias, as variations in disease prevalence, the distribution of lesion severity, and image quality filtering or exclusion criteria can inflate apparent performance and reduce generalizability to routine clinical settings. Given the substantial residual heterogeneity after modality stratification, future work should incorporate influence and outlier diagnostics to identify heterogeneity driving studies, including leave one out analyses in the bivariate random effects model and hierarchical influence checks to detect high leverage studies. In addition, reporting prediction ranges for sensitivity and specificity will better communicate expected performance variability in new settings rather than only the mean pooled estimate. To improve comparability and reproducibility, we propose minimum standards: reporting key acquisition parameters and quality control criteria; transparent annotation procedures (definitions/thresholds, labeling level, annotator credentials, agreement, and adjudication); prespecified data splits to avoid leakage with reported operating thresholds; explicit reporting of the threshold strategy per study (fixed vs. optimized, and whether optimization was validation-based vs. test set-based), with specific disclosure of any test set-optimized thresholds that may inflate performance; and clear justification for pooled group comparisons using DeLong or Z tests under threshold variation and study-level heterogeneity, or the use of hierarchical approaches (e.g., meta-regression or interaction models) that explicitly account for these sources of variability; and external validation with transparent reporting and, where feasible, code/model sharing. From a technical perspective, several improvements may further enhance performance: boundary-preserving mask heads (e.g., BMask R-CNN) [67], deformable or cascade detection heads for Faster R-CNN [68], and 3-D extensions for volumetric CBCT when applicable [24]. Together, these avenues can point toward faster, safer, and more generalizable caries detection systems.

## 5. Conclusions

Faster R-CNN and Mask R-CNN show promise for dental caries detection, but the current evidence is limited by substantial heterogeneity, predominantly retrospective designs, and variable imaging and labeling practices. Across the included studies, Mask R-CNN yielded higher pooled performance estimates overall, with the clearest differences in radiographic workflows; however, these findings arise from indirect comparisons and should be interpreted as suggestive rather than definitive given between-study heterogeneity and uncertainty in the reference standard. In photographic images, differences in pooled sensitivity and specificity were not statistically significant, which may reflect variability in image quality, limited standardization, and smaller or less comparable datasets. Overall, these models may serve as adjunctive decision-support tools, but stronger conclusions will require prospective, multi-center studies with standardized acquisition and annotation, prespecified analysis plans, and independent external validation, alongside sensitivity and meta-regression analyses to evaluate the stability of observed performance differences.

## Figures and Tables

**Table 1:** Characteristics of Included Studies.

Authors/Year	Machine Learning Algorithms	Number of Annotators/Qualifications	Consensus Approach	Blinding	Caries Definition
N. Cauás et al., 2023 [32]	YOLOv5 and Faster R-CNN	Manually annotated	Not reported	Not reported	Not reported
X. T. Chen et al., 2022 [33]	Faster R-CNN	2 endodontist and 1 radiologist	Independent labeling; disagreements resolved by discussion	Not reported	Radiolucent area between adjacent contacts with specified radiographic appearance
M. Estai et al., 2022 [34]	Faster R-CNN and Inception-ResNet-v2	3 qualified dentists	2 dentists draw rectangles; consensus required. Discrepancies resolved by 3rd dentist	2 dentists were blinded to each other during initial review	Detection per WHO standard + ICDAS
S. Fan et al., 2023 [35]	YOLO V5, Faster R-CNN, Retinanet	Manually annotated	Not reported	Not reported	Artificial demineralization model on 30 bovine teeth with “score1, score2, score3” groups (3 phases).
A. Juyal et al., 2023 [36]	YOLOv3, Faster R-CNN	Not reported	Not reported	Not reported	“Dental cavities/caries” in camera images, no operational definition or grading stated.
L. Kunt et al., 2023 [37]	YOLOv5, Faster R-CNN, RetinaNet, EfficientDet;	1 specialist in cariology	No consensus (single annotator)	Not reported	7257 “carious lesions” annotated on bitewing radiographs using minimal bounding boxes.
Mahaveerakannan R et al., 2024 [38]	YOLOv3, Faster R-CNN, RetinaNet, SSD	1 qualified dentist	No consensus (single annotator)	Not reported	ICCMS-based classes: 0 sound (NSC), 1 visually non-cavitated (VNC), 2 cavitated (moderate), 3 late cavitated (extensive)
E. Y. Park et al., 2022 [39]	U-Net, ResNet-18, Faster R-CNN	1 board-certified dentist	No consensus (single annotator)	Not reported	Ground truth per ICDAS; only distinct caries ICDAS codes 4–6 annotated as “caries cases”
M. T. G. Thanh et al., 2022 [40]	Faster R-CNN, YOLOv3, RetinaNet, SSD	1 experienced dentist	No consensus (single annotator)	Not reported	ICCMS-based classes: 0 sound (NSC), 1 VNC, 2 cavitated with localized enamel breakdown or dentin shadow, 3 late cavitated with visible dentin
J. Velusamy et al., 2024 [41]	Faster R-CNN, YOLOv3	Not reported	Not reported	Not reported	“Three distinct caries level” (no explicit operational definition provided)
Yuang Zhu et al., 2022 [42]	Faster-RCNN	1 doctor	No consensus (single annotator)	Not reported	Not reported (caries labeled as “caries positions/locations,” no case definition stated)
E. T. Chaves 2024 [43]	Mask R-CNN	2 graduate students; 3 PhD students; 2 caries experts	Final annotations in joint sessions; disagreements resolved by consensus	Patient-level de-identification: radiographs de-identified; database contained only images	“Primary caries lesions” on tooth surfaces and “secondary caries around restorations” on bitewings
Yanbin Guo 2024 [44]	Mask R-CNN	3 experienced dentists	Not reported	Not reported	“Non-normal teeth” includes caries; dataset categories include “caries” and other abnormalities (residual root, retainer, filling, etc.), later merged into “abnormal” for training
K. Moutselos 2019 [45]	Mask R-CNN	2 ICDAS dental experts	Explicit consensus noted for superpixel-based lesion segmentation settings (two ICDAS experts)	Not reported	Occlusal caries detection and classification across full ICDAS 7-class scale (0–6) on intraoral images
N. van Nistelrooij 2024 [46]	Mask R-CNN	2 PhD students, 3 senior dentists, 1 caries expert	Discrepancies resolved via joint discussions	Not reported	Severity score based on lesion key points: 0.0 = lesion not reaching dentine, 1.0 = lesion reaching pulp (staging of secondary caries)
Lizheng Liu 2020 [47]	Mask R-CNN	Not reported	Not reported	Not reported	Caries operationalized as “decayed tooth” among 7 disease classes
Umer Rashid 2022 [48]	Mask R-CNN	2 qualified dentists	comparing 2 dentist labeling correctness	Not reported	Defines dental cavity as “destruction of a tooth’s tissue”

**Table 2:** Performance metrics of Faster R-CNN papers.

Authors/Year	Country	Imaging Modality	N	TP	TN	FP	FN	Accuracy	Sensitivity (95% CI)	Specificity (95% CI)	Precision	F1 Score	ROC AUC
N. Cauás et al., 2023 [32]	Brazil	dental radiographs	294	42	206	15	31	0.84	0.575 (0.50–0.65)	0.93 (0.87–0.97)	0.73	0.65	0.752
X. T. Chen et al., 2022 [33]	China	bitewing	2087	444	1363	111	169	0.87	0.724 (0.69–0.76)	0.925 (0.91–0.94)	0.8	0.76	0.824
M. Estai et al., 2022 [34]	Australia	bitewing	2468	680	1465	239	84	0.87	0.89 (0.87–0.91)	0.86 (0.84–0.88)	0.74	0.81	0.875
S. Fan et al., 2023 [35]	China	optical coherence tomography	100	31	53	4	12	0.85	0.72 (0.65–0.79)	0.93 (0.87–0.97)	0.88	0.79	0.82
A. Juyal et al., 2023 [36]	India	intraoral photos	300	113	113	32	42	0.75	0.73 (0.65–0.81)	0.78 (0.70–0.86)	0.78	0.76	0.76
L. Kunt et al., 2023 [37]	Czech Republic	bitewing	598	53	497	32	16	0.92	0.77 (0.72–0.82)	0.94 (0.88–0.97)	0.62	0.69	0.855
Mahaveerakannan R et al., 2024 [38]	India	intraoral photos	5630	785	4271	273	301	0.90	0.723 (0.65–0.79)	0.94 (0.88–0.98)	0.742	0.73	0.831
E. Y. Park et al., 2022 [39]	South Korea	intraoral photographic images	2348	546	1428	182	192	0.84	0.74 (0.68–0.80)	0.887 (0.83–0.93)	0.75	0.745	0.814
M. T. G. Thanh et al., 2022 [40]	Vietnam, Japan	intraoral photos	750	136	519	40	55	0.87	0.712 (0.64–0.78)	0.929 (0.87–0.97)	0.773	0.741	0.82
J. Velusamy et al., 2024 [41]	India	panoramic	600	317	201	47	35	0.86	0.90 (0.85–0.95)	0.81 (0.75–0.87)	0.87	0.88	0.86
Yuang Zhu et al., 2022 [42]	China	periapical	800	203	482	36	79	0.86	0.72 (0.65–0.79)	0.93 (0.87–0.97)	0.85	0.78	0.83

**Table 3:** Performance metrics of Mask R-CNN papers.

Authors/Year	Country	Imaging Modality	N	TP	TN	FP	FN	Accuracy	Sensitivity (95% CI)	Specificity (95% CI)	Precision	F1 Score	ROC AUC
E. T. Chaves 2024 (Primary caries) [43]	Netherlands Brazil Germany	bitewing	12,750	193	12,404	86	67	0.988	0.742 (0.68–0.82)	0.99 (0.98–0.99)	0.687	0.712	0.849
E. T. Chaves 2024 (Secondary caries) [43]	Netherlands Brazil Germany	bitewing	12,750	377	12,068	160	145	0.976	0.722 (0.622–0.782)	0.987 (0.98–0.99)	0.702	0.713	0.804
Yanbin Guo 2024 [44]	China USA	panoramic	1008	733	150	25	100	0.876	0.879 (0.86–0.90)	0.857 (0.81–0.91)	0.967	0.921	0.868
K. Moutselos 2019 [45]	Greece	Intraoral images	4909	2539	846	339	1185	0.691	0.682 (0.67–0.70)	0.714 (0.69–0.74)	0.882	0.767	0.698
N. van Nistelrooij 2024 (all lesions) [46]	Netherlands Germany Denmark	bitewing	2612	282	2147	78	105	0.932	0.729 (0.69–0.78)	0.966 (0.96–0.97)	0.783	0.755	0.851
N. van Nistelrooij 2024 (dentine lesions) [46]	Netherlands Germany Denmark	bitewing	2612	246	2240	79	47	0.951	0.839 (0.80–0.88)	0.964 (0.96–0.97)	0.757	0.796	0.902
Lizheng Liu 2020 [47]	China Sweden	Intraoral images	2556	634	1660	178	84	0.898	0.883 (0.85–0.92)	0.903 (0.83–0.97)	0.780	0.828	0.893
Umer Rashid 2022 (photographic images) [48]	Pakistan United Kingdom	photographic images	90	71	10	1	8	0.90	0.90 (0.85–0.95)	0.91 (0.84–0.94)	0.986	0.940	0.903
Umer Rashid 2022 (radiographs) [48]	Pakistan United Kingdom	X-ray radiographs	936	881	41	5	9	0.985	0.989 (0.97–1.00)	0.891 (0.84–0.95)	0.994	0.992	0.946
Umer Rashid 2022 (mixed set) [48]	Pakistan United Kingdom	photographic images, X-ray radiographs	210	139	47	4	20	0.886	0.874 (0.81–0.92)	0.922 (0.87–0.98)	0.972	0.920	0.898

**Table 4:** Meta-Regression of Faster R-CNN and Mask R-CNN in detecting dental caries.

Covariate	Sub- Group	Sensitivity (95% CI)	*p*-Value	Specificity (95% CI)	*p*-Value	AUC	*p*-Value	I^2^ (Sens/Spec)	τ^2^ (Sens/Spec)
All images	Faster R-CNN	71.7% (62%; 79.7%)	0.0244 *	81.4% (74.8%; 86.6%)	0.00089 ***	0.84	0.0053 **	96.8%/97.9%	0.297/0.422
	Mask R-CNN	85.6% (75.5%; 92%)		94.2% (87.9%; 97.3%)		0.95		97.5%/99.5%	0.484/2.577
Radiographic images	Faster R-CNN	67.2% (51.3%; 79.9%)	0.0497 *	85% (78.5%; 89.8%)	0.00105 **	0.86	0.0067 **	97.2%/95.7%	0.405/0.327
	Mask R-CNN	86.3% (68.4%; 94.9%)		96.5% (90.8%; 98.7%)		0.97		96.9%/98.2%	0.547/0.885
Photographic images	Faster R-CNN	77.3% (66.9%; 85.2%)	0.435	75.1% (64.6%; 83.3%)	0.156	0.83	0.048 *	96.8%/94.6%	0.437/0.128
	Mask R-CNN	83.5% (67.3%; 92.5%)		86% (70.5%; 94.1%)		0.91		98.3%/98.8%	0.792/0.856

AUC, area under the curve. * *p* < 0.05. ** *p* < 0.01. *** *p* < 0.001.

## Data Availability

The data presented in this study were extracted from published studies cited in the reference list. The extracted dataset and analytical files are available from the corresponding author upon reasonable request due to the secondary nature of the data and copyright considerations.

## Supplementary Materials

The following supporting information can be downloaded at: https://www.mdpi.com/article/10.3390/diagnostics16050731/s1, Table S1: Quality Assessment of Diagnostic Accuracy Studies-AI (QUADAS-AI); Table S2: Radiomics Quality Score (RQS); File S1: The full database-specific strategies; File S2: Details regarding the imaging types, image resolution, equipment, camera settings, and standardization processes for each paper; FileS3: PRISMA 2020 Checklist.
