# Supplementary material for: Deep Learning-Based Dental Caries Diagnosis: A Modality-Stratified Systematic Review and Meta-Analysis of Faster R-CNN and Mask R-CNN
Source: Diagnostics (Basel). 2026 Mar 1;16(5):731. doi: 10.3390/diagnostics16050731 (PMC12985255; doi:10.3390/diagnostics16050731)
Supplement: Supplementary file 1 [file diagnostics-16-00731-s001.zip › diagnostics-4119562-supplementary File S1.pdf]

Database	Topic	Results	Search term
**Pubmed** June 15, 2025	Faster R-CNN	132	((oral OR tooth OR mouth OR teeth OR dental OR "tooth disease" OR "mouth disease" OR "dental caries" OR "periodontal disease" OR "periodontitis" OR "endodontics" OR "tooth fracture" OR "tooth periapical disease" OR "tooth crown" OR "prosthodontics" OR "tooth abscess" OR "tooth impaction")) AND (Convolutional neural networks OR CNN OR Recurrent convolutional neural networks OR RCNN OR "Faster Recurrent convolutional neural networks OR Faster R-CNN OR Faster RCNN)
	Mask R-CNN	84	((oral OR tooth OR mouth OR teeth OR dental OR "tooth disease" OR "mouth disease" OR "dental caries" OR "periodontal disease" OR "periodontitis" OR "endodontics" OR "tooth fracture" OR "tooth periapical disease" OR "tooth crown" OR "prosthodontics" OR "tooth abscess" OR "tooth impaction")) AND (Convolutional neural networks OR CNN OR Recurrent convolutional neural networks OR RCNN OR "Mask Recurrent convolutional neural networks OR Mask R-CNN OR Mask RCNN)
**Embase** June 15, 2025	Faster R-CNN	82	(‘oral'/exp OR ‘tooth'/exp OR ‘mouth'/exp OR ‘teeth'/exp OR ‘dental'/exp OR ‘tooth disease'/exp OR ‘mouth disease'/exp OR ‘dental caries'/exp OR ‘periodontal disease'/exp OR ‘periodontitis'/exp OR ‘endodontics'/exp OR ‘tooth fracture'/exp OR ‘tooth periapical disease'/exp OR ‘tooth crown'/exp OR ‘prosthodontics'/exp OR ‘tooth abscess'/exp OR ‘tooth impaction'/exp) AND (‘Convolutional neural networks'/exp OR ‘CNN'/exp OR ‘Recurrent convolutional neural networks'/exp OR ‘RCNN'/exp OR ‘Faster Recurrent convolutional neural networks'/exp OR ‘Faster R-CNN'/exp OR ‘Faster RCNN'/exp)
	Mask R-CNN	70	(‘oral'/exp OR ‘tooth'/exp OR ‘mouth'/exp OR ‘teeth'/exp OR ‘dental'/exp OR ‘tooth disease'/exp OR ‘mouth disease'/exp OR ‘dental caries'/exp OR ‘periodontal disease'/exp OR ‘periodontitis'/exp OR ‘endodontics'/exp OR ‘tooth fracture'/exp OR ‘tooth periapical disease'/exp OR ‘tooth crown'/exp OR ‘prosthodontics'/exp OR ‘tooth abscess'/exp OR ‘tooth impaction'/exp) AND (‘Convolutional neural networks'/exp OR ‘CNN'/exp OR ‘Recurrent convolutional neural networks'/exp OR ‘RCNN'/exp OR ‘Mask Recurrent convolutional neural networks'/exp OR ‘Mask R-CNN'/exp OR ‘Mask RCNN'/exp)
**Scopus** June 15, 2025	Faster R-CNN	285	(oral OR tooth OR mouth OR teeth OR dental OR "tooth disease" OR "mouth disease" OR "dental caries" OR "periodontal disease" OR "periodontitis" OR "endodontics" OR "tooth fracture" OR "tooth periapical disease" OR "tooth crown" OR "prosthodontics" OR "tooth abscess" OR "tooth impaction") AND (Convolutional neural networks OR CNN OR Recurrent convolutional neural networks OR RCNN OR "Faster Recurrent convolutional neural networks OR Faster R-CNN OR Faster RCNN)
	Mask R-CNN	155	(oral OR tooth OR mouth OR teeth OR dental OR "tooth disease" OR "mouth disease" OR "dental caries" OR "periodontal disease" OR "periodontitis" OR "endodontics" OR "tooth fracture" OR "tooth periapical disease" OR "tooth crown" OR "prosthodontics" OR "tooth abscess" OR "tooth impaction") AND (Convolutional neural networks OR CNN OR Recurrent convolutional neural networks OR RCNN OR "Mask Recurrent convolutional neural networks OR Mask R-CNN OR Mask RCNN)
**Web of Science**	Faster R-CNN	117	((oral OR tooth OR mouth OR teeth OR dental OR "tooth disease" OR "mouth disease" OR "dental caries" OR "periodontal disease" OR "periodontitis" OR "endodontics" OR

June 15, 2025			"tooth fracture" OR "tooth periapical disease" OR "tooth crown" OR "prosthodontics" OR "tooth abscess" OR "tooth impaction")) AND (Convolutional neural networks OR CNN OR Recurrent convolutional neural networks OR RCNN OR "Mask Recurrent convolutional neural networks OR Mask R-CNN OR Mask RCNN)
	Mask R-CNN	106	((oral OR tooth OR mouth OR teeth OR dental OR "tooth disease" OR "mouth disease" OR "dental caries" OR "periodontal disease" OR "periodontitis" OR "endodontics" OR "tooth fracture" OR "tooth periapical disease" OR "tooth crown" OR "prosthodontics" OR "tooth abscess" OR "tooth impaction")) AND (Convolutional neural networks OR CNN OR Recurrent convolutional neural networks OR RCNN OR "Mask Recurrent convolutional neural networks OR Mask R-CNN OR Mask RCNN)
