# Supplementary material for: Deep Learning-Based Dental Caries Diagnosis: A Modality-Stratified Systematic Review and Meta-Analysis of Faster R-CNN and Mask R-CNN
Source: Diagnostics (Basel). 2026 Mar 1;16(5):731. doi: 10.3390/diagnostics16050731 (PMC12985255; doi:10.3390/diagnostics16050731)
Supplement: Supplementary file 1 [file diagnostics-16-00731-s001.zip › diagnostics-4119562-supplementary File S2.pdf]

Authors	Imaging Type	Image Resolution	Equipment	Camera Settings	Standardization Processes
N. Cauás et al 2023	Dental radiographs	748x512 pixels (RGB)	Not specified (general X-ray radiographs)	Not specified	Resizing, normalization, augmentation (e.g., flipping, rotation); dataset split (85% training, 15% validation); limited data handling (20%-100% subsets)
X. T. Chen et al 2022	Bitewing	(960–980) × (750–755) pixels (8-bit JPG)	Gendex expert DC system by Gendex Dental Systems (USA)	65 kV, 7 mA, exposure time 0.125–0.32 s	Random split (training/validation 818 images, test 160); brightness/contrast adjustment; no resizing mentioned
M. Estai et al 2022	Bitewing	994 × 761 to 2130 × 1490 pixels (PNG)	Photostimulable phosphor plate technique	Not specified	Resizing to 640 × 480 pixels; augmentation (rotation, shift, scaling, flip); 10-fold cross-validation; normalization
S. Fan et al 2023	Optical Coherence Tomography (OCT) images	6mm × 6mm field of view; imaging depth 4.53 mm	Swept-Source OCT (SS-OCT) system by Santec HSL-20 laser (Japan)	Central wavelength 1310 nm; bandwidth 105 nm; sweep rate 100 kHz; coherence length 16 mm	Manual annotation; dataset split (training/testing); no specific resizing/augmentation mentioned
A. Juyal et al 2023	Intraoral photos	Not specified	Not specified	Not specified	Dataset split (250 training, 50 testing); labeling with LabelImg; no specific standardization mentioned
L. Kunt et al 2023	Bitewing	Rescaled to 896 × 1024 pixels	Various intraoral X-ray units (sensor sizes 31×41 mm to 27×54 mm)	Not specified	Rescaling to uniform 896×1024; augmentation (flip, translation, rotation, blur, gamma correction); dataset split (70% training, 15% validation, 15% test)
Mahaveerakannan R et al 2024	Intraoral photos	Not specified (JPEG format)	iPhone 7 by Apple (USA)	3 views (central, right/left lateral); low-speed handpiece for cleaning	Resizing to uniform; augmentation (flipping, rotation); dataset split (1902 training, 750 testing); normalization
E. Y. Park et al 2022	Intraoral photographic images	1280 × 720 pixels (RGB)	Qraypen professional intraoral camera by	Not specified	Random split (1638 training, 410 validation, 300 test); augmentation (shifting,

			AIOBIO (South Korea)		symmetry, blurring); pixel-wise labeling
M. T. G. Thanh et al 2022	Intraoral photos	Not specified (JPEG)	iPhone 7 by Apple (USA)	3 views (central, right/left lateral); cleaning with low-speed handpiece	Resizing to uniform; augmentation (flipping, rotation); dataset split (1902 training, 750 testing); normalization
J. Velusamy et al 2024	Panorami c	512 × 512 pixels	Not specified	Not specified	Resizing to 512×512; augmentation (brightness/contrast change, flip); dataset split (training/validation/test)
Yuang Zhu et al 2022	Periapical	Not specified	Not specified	Not specified	Data augmentation (brightness/contrast change, flip); resizing to uniform; dataset split (training/testing)
E. T. Chaves 2024	bitewing	Not specified	Not specified	Not specified	Data augmentation (flipping, resizing, cropping); ten-fold cross-validation; resizing during inference
Yanbin Guo 2024	panorami c	Not specified	Not specified	Not specified	Data augmentation (panning, scaling); random split (908 training, 100 validation)
K. Moutselos 2019	Intraoral images	1024x768 (Height x Width)	Carestream 1200 intraoral camera	Focal distance 3-25 mm; 6 white LEDs; USB 2.0	Superpixel segmentation (jSLIC); transfer learning; augmentation (flip, rotation, shear)
N. van Nistelrooij 2024	bitewing	Not specified	Not specified	Not specified	Data augmentation (flipping, resizing, cropping); ten-fold cross-validation
Lizheng Liu 2020	Intraoral images	Not specified	Not specified	Not specified	Semi-automatic labeling; data collection from clinics; augmentation not specified
Umer Rashid 2022	Photograp hic images, X-ray radiograp hs	Not specified	Not specified	Canon EOS 7D for photos; various X-ray from Pakistan	Histogram equalization; manual annotation; dataset split (70:30 or 80:20)
