# Supplementary material for: Deep Learning-Based Dental Caries Diagnosis: A Modality-Stratified Systematic Review and Meta-Analysis of Faster R-CNN and Mask R-CNN
Source: Diagnostics (Basel). 2026 Mar 1;16(5):731. doi: 10.3390/diagnostics16050731 (PMC12985255; doi:10.3390/diagnostics16050731)
Supplement: Supplementary file 1 [file diagnostics-16-00731-s001.zip › diagnostics-4119562-supplementary tables.pdf]

Table S1. Quality Assessment of Diagnostic Accuracy Studies-AI (QUADAS-AI) Risk of bias summary: review authors' judgments about each risk of bias items

Paper		Risk of bias				Applicability concerns		
		**Patient Selection**	**Index Test**	**Reference Standard**	**Flow and Timing**	**Patient Selection**	**Index Test**	**Reference Standard**
Faster R-CNN	N. Cauás et al	x	+	x	+	+	+	+
	X. T. Chen et al	x	+	+	+	x	+	+
	M. Estai et al	x	+	+	+	x	+	x
	S. Fan et al	x	+	-	+	x	+	x
	A. Juyal et al et al	-	+	x	x	-	x	x
	L. Kunt et al	x	+	+	+	x	+	+
	Mahaveerakannan R et al	-	+	x	x	x	+	x
	E. Y. Park et al	+	+	-	+	+	+	+
	M. T. G. Thanh et al	x	+	-	+	x	+	+
	J. Velusamy et al	x	+	x	x	-	+	-
	Yuang Zhu et al	-	+	x	+	x	+	x
Mask R-CNN	E. T. Chaves	+	+	+	+	+	+	+
	Yanbin Guo	x	+	+	x	+	+	x
	K. Moutselos	x	+	x	+	x	+	+
	N. van Nistelrooij	x	+	+	+	x	+	+
	Lizheng Liu	-	+	x	+	x	+	+
	Umer Rashid	x	+	x	+	x	+	x

(+) low risk, **(x)** unclear risk, **(-)** high risk.

Table S2. Radiomics Quality Score (RQS)
(1) Image Protocols (2) Multiple Segmentations (3) Phantom study (4) Imaging at multiple time points (5) Feature reduction or adjustment for multiple testing (6) Multivariable analysis (7) Biological correlates (8) Cut-off analysis (9) Discrimination statistics (10) Calibration statistics (11) Prospective study (12) Validation (13) Comparison to 'gold standard' (14) Potential clinical applications (15) Cost effectiveness analysis (16) Open science and data

	**1 (1-2)**	**2 (0–1)**	**3 (0–1)**	**4 (0–1)**	**5 (-3–3)**	**6 (0–1)**	**7 (0–1)**	**8 (0–1)**	**9 (1–2)**	**10 (1–2)**	**11 (0–7)**	**12 (-5–5)**	**13 (0–2)**	**14 (0–2)**	**15 (0–1)**	**16 (1–4)**	**Total**
N. Cauás 2023	2	0	1	1	1	1	1	1	2	1	5	2	2	2	0	2	24

X.T. Chen 2022	2	0	1	1	3	1	0	1	2	1	6	4	2	2	1	2	29
M. Estai 2022	1	0	1	1	1	1	1	1	2	1	4	3	1	2	0	2	22
L. Kunt 2023	2	0	1	1	2	1	1	1	2	1	5	4	2	2	1	3	29
Velusamy 2024	1	0	0	1	1	1	1	1	2	1	4	2	1	2	0	2	20
Yuang Zhu 2022	2	0	1	1	3	1	1	1	2	1	5	4	2	2	0	2	28
E. Chaves 2024	2	1	1	1	3	1	1	1	2	2	6	4	2	2	1	3	33
Y. Guo 2024	2	1	1	1	2	1	0	1	2	1	5	2	2	2	0	2	25
N. van Nistelrooij 2024	2	1	1	1	3	1	1	1	2	1	6	4	2	2	1	2	31
U. Rashid 2022	2	1	1	1	2	1	1	1	2	1	4	3	2	2	1	2	27
Total	18/20	4/10	9/10	10/10	21/30	10/10	8/10	10/10	20/20	11/20	50/70	32/50	18/20	20/20	5/10	22/40	268/360
